# Lifespan and associated factors of peripheral intravenous Cannula among infants admitted in public hospitals of Mekelle City, Tigray, Ethiopia, 2016

**DOI:** 10.1186/s12912-017-0227-1

**Published:** 2017-06-15

**Authors:** Eskedar Birhane, Kalayou Kidanu, Mekuria Kassa, Dawit Gerezgiher, Lidia Tsegay, Brhanu Weldu, Genet Kidane, Hadgu Gerensea

**Affiliations:** 1 School of Nursing, College of Health Sciences and Referral Hospital, Axum University, Axum, Ethiopia; 2 Department of Nursing, College of Health Sciences, Mekelle University, Mekelle, Ethiopia; 3 School of Medicine, College of Health Sciences and Referral Hospital, Axum University, Axum, Ethiopia; 4 Department of Public Health, College of Health Sciences, Mekelle University, Mekelle, Ethiopia

**Keywords:** Lifespan, Associated factor, Peripheral intravenous cannula, Infant

## Abstract

**Background:**

Peripheral Intravenous cannula (IV) is the most common vascular access device used to administer medications with the exception of medication or fluid with high or low PH or hyperosmolarity which may cause severe damage to small veins. The insertion of a peripheral intravenous cannula in newborn infants can be difficult. Appropriate veins with sufficient capacity to insert a cannula become less available throughout the hospital stay. Once a peripheral intravenous cannula is inserted, it is desirable that its patency can be maintained as long as possible. This study was aimed to assess the lifespan and associated factors of peripheral intravenous cannula among infants admitted in public hospitals of Mekelle city, Tigray, Ethiopia, 2016.

**Methods:**

The method used was a prospective cohort study. 178 study subjects were recruited using systematic random sampling technique. The data was collected by structured questionairre and observational checklist.

**Results:**

More than half of infants (94) had a short cannula lifespan (below 30 h). Multivariable logistic regression analysis showed that Pediatric intensive care unit (PICU) [AOR = 6.93; 95% CI (1.56,30.71)], clinical experience (3-5 years) [AOR = 0.168; 95% CI (0.060-0.469)], insertion site (arm) [AOR = 0.126;95% CI (0.046-0.349)], reason for removal (dislodgement and complication) [AOR = 8.15; 95% CI (2.49,26.63) [AOR = 10.48;95% CI (3.08,35.65)], medication [AOR = 0.17;95% CI (0.37,0.784)], corticosteroids [0.164; 95% CI (0.034,0.793)] and blood transfusion [AOR = 0.12; 95% CI (0.028-0.509)] were the statistically significant variables associated with the lifespan of a peripheral intravenous cannula.

**Conclusions:**

Untimely removal of peripheral intravenous cannulas was higher in infants and demographic. Cannulation and health care factors had significant effects on the lifespan of a peripheral intravenous cannula.

## Background

Intravenous (IV) therapy relates to the administration of therapeutic solutions directly into a vein. Compared with other approaches such as intramuscular injection, intravenous (IV) therapy is the fastest and most reliable way to deliver fluids or medications [1]. With more than 90% of hospitalized patients requiring IV therapy, the proportion of patients requiring infusion devices has increased significantly over the past 30 years. IV therapy, which has multiple indications, can maintain the fluid and electrolyte balance of the body, infuse medications, transfuse blood or blood components and provide some nutritional support such as total parenteral nutrition [2].

The lifespan of a peripheral intravenous cannula is an important issue, and its survival depends on many variables, including the size, frequency of usage, methods of administration of medications and fluids, and others. It is noticed that when certain drugs are administered, the peripheral intravenous cannula is infiltrated faster. Factors affecting the life span of peripheral intravenous cannula have been studied in newborns and infants [3, 4]. Heparin and limb splinting have been used in an attempt to prolong duration of the peripheral intravenous cannula [5, 6].

Although peripheral intravenous cannulas are routinely used in hospital admitted infants, the risk of complications remains high. Non-elective removal of a peripheral intravenous cannula as a result of complications occurs in up to 78% of insertions and can lead to untimely removal of up to 95% of the devices [7]. It is difficult to ascertain precise complication rates due to a lack of consistent definition for complications, significant variation in reporting among different facilities, and studies focused on specific complications. Routine replacement of peripheral intravenous cannulas is not recommended in neonates given challenges associated with their small, fragile veins and limited vascular access [8, 9]. Peripheral intravenous cannulas that are left in situ when they are no longer required may predispose the patient to intravascular infection. There is also emerging evidence that the regular refitting of peripheral intravenous cannulas as a strategy to reduce intravascular infection provides no protective benefit, and if removed when clinically indicated, can avoid millions of cannula insertions which can substantially reduce health care costs and improve patient satisfaction [10].

The average lifespan of peripheral intravenous cannulas in neonates and infants reported in the literature ranges from 15 to 54 h and 12 to 274 h respectively. Cannulas can be safely maintained with adequate monitoring for up to 144 h in critically ill children [11, 12]. Accessing the vascular system necessitates penetration of the skin and stabilization of the cannula in situ. The lifespan of peripheral intravenous cannula and its survival depends on many factors, including the size cannula, frequency of usage, methods of administration of medications, increased number of attempts at placement of intravenous cannulas, fluids and others [3, 4].

Insertion of peripheral intravenous cannulas is a painful procedure for infants, therefore, identifying which factors affect the lifespan of a peripheral intravenous cannula will help reduce complications and increase duration of cannula patency. Additional issues that must be considered include the potential for prolonged hospital stay as a result of cannula-associated complications, delays in therapy, and the impact on patient/family satisfaction. Thus, reducing complications will lead to a reduction in downstream activity, as well as the time associated with the reassessment of a failed peripheral intravenous cannula, reducing additional supplies for the peripheral intravenous cannula replacement, and reducing the risk of needle stick injury.

This study evaluates the lifespan of peripheral intravenous cannulas in infants and identifies factors which affect the lifespan of peripheral intravenous cannula that contribute to the current knowledge regarding intravenous practices in infants.

## Methods

The study was conducted in Mekelle, Ethiopia, an area with two governmental hospitals and three private hospitals. Ayder Hospital, a major referral and teaching hospital in the Tigray region, opened in 2007 with 500 beds, serving 32,000 patients. The second, Mekelle Hospital, a regional referral and teaching hospital, was established in 1962 with 162 beds and a total annual flow of 4276 patients. The study period spanned from October, 2015 to May, 2016. The study design was an institutional based cross-sectional study design.

### Sample size determination and sampling procedures

A total of 178 samples were employed among the two hospitals using systematic random sampling.

### Data collection procedure and tool

Data was collected by observational checklist and structured questionnaire. Continuous follow up and supervision by the supervisors and principal investigators occurred throughout the data collection period.

### Data quality assurance, entry and analysis

The data was recorded, cleaned and analyzed using Statistical Package for Social Sciences (SPSS) version 20 software statistical packages. Frequencies and proportions were used to describe the study population in relation to relevant variables. Logistic regression was computed to assess statistical association via calculating Crud Odds and Adjusted odds ratio to see the influence of independent variables on dependent variables, and significance of statistical association was assured or tested using 95% confidence interval and *P*-value (<0.05).

### Ethical consideration

Ethical clearance was secured from the Mekelle University, College of Health Science research review committee. An official letter of permission was obtained from Tigray Regional Health Bureau, Ayder Referral Hospital, and Mekelle Hospital medical director offices. Respondents were well informed about the purpose of the study, and information was collected after oral and written consent from each nurse was obtained. Furthermore, oral consent was obtained from parents\guardians, primarily mothers which is approved by Ethical Review board (IRB) committee of mekelle university. Since no intervention was given written consent was not mandatory. The data was collected only through observation using checklist. Information was recorded anonymously and confidentially, and beneficence was assured throughout the study period.

## Result

### I. Demographic and clinical data of infants

A total of 178 infants with peripheral intravenous cannulas were selected. Infant ages ranged from one day to 11 months of age with a median and inter quartile range (2 ± 5.7). Weight ranged from 1 to 11.5 kgs (median 4.0 ± 3.46). One hundred six of the participants were males (59.6%). Regarding to admission of infants in pediatric units, 101 (56.7%) infants were admitted in the pediatric ward. One hundred fifty-eight (88.8%) of infants were acutely sick (Table 1).

**Table 1:** Distribution of demographic and clinical data by lifespan of peripheral intravenous cannula, among infants admitted in public hospitals of Mekelle city, Tigray, Ethiopia 2016

Variables	Category	Frequency (%)	Lifespan of PIVC*	
			Short long	Lifespan lifespan
Age	Neonate	57 (32)	35	22
	Infant	121 (68)	59	62
Sex	Female	72 (40.4)	41	31
	Male	106 (59.6)	53	53
Weight (in kg)	<1.499	9 (5.1)	05	04
	1.5-2.499	28 (15.7)	20	08
	2.5-3.499	29 (16.3)	18	11
	>3.5	112 (62.9)	51	61
Pediatric unit	NICU^a^	57 (32)	35	22
	PICU^b^	20 (11.2)	15	05
	PW^c^	101 (56.7)	44	57
Sickness	Chronic	20 (11.2)	10	10
	Acute	158 (88.8)	84	74

^a^ *NICU* Neonatal intensive care unit, ^b^ *PICU* Pediatric intensive care unit, ^c^ *PW* Pediatric ward, *PIVC Peripheral intravenous cannula

### II. Cannulation characteristics

Complete data regarding cannula size was obtained for 178 peripheral intravenous cannula insertions. Insertion was accomplished for 175 infants (98.3%) by 24 gauge cannulas. More than two-thirds, 118 (66.3%) cannulas were inserted in veins which were visible but not palpable. When looking at the entire sample, 77 (43.3%) of cannulas were inserted into veins of the scalp. About 88% of the peripheral intravenous cannulas were placed for administration of medication with or without fluid and blood transfusion. The majority of peripheral intravenous cannulas 63 (35.4%) was discontinued due to dislodgement (Table 2).

**Table 2:** Cannulation Characteristics of infants by lifespan of peripheral intravenous cannula admitted in public hospitals of Mekelle city, Tigray, Ethiopia, 2016

Variables	Category	Frequency (%)	Lifespan of PIVC	
			Short lifespan	Long lifespan
Cannula size/gauge	24 gauge	175 (98.3)	91	84
	22 gauge	3 (1.7)	03	00
Site of insertion	Scalp	77 (43.3)	48	29
	Arm	54 (30.3)	13	41
	Hand	29 (16.3)	21	08
	Leg	18 (10.1)	12	06
Reason for removal	DCWC^a^	42 (23.6)	09	33
	Dislodged	63 (35.4)	37	26
	Complication	51 (28.7)	36	15
	Blocked	22 (12.4)	12	10
Venous condition	Visible but not palpable	118 (66.3)	65	53
	Not visible and palpable	60 (33.7)	29	31
Medication	No	21 (11.8)	16	05
	Yes	157 (88.2)	78	79
Fluid	No	40 (22.5)	19	21
	Yes	138 (77.5)	75	63
Blood Transfusion	No	161 (90.4)	89	72
	Yes	17 (9.6)	05	12

^a^ *DCWC* Discontinue without complication

### III. Health care related factors

Based on the data received, of the total sampled infants with peripheral intravenous cannula, in 78 cases (43.8%) insertion was accomplished in 2–3 attempts, with median and inter quartile range (2 ± 1), ranges from 1 to 6 attempts. Antibiotics were among the most commonly administered medications accounting for 141 (79.2%) of peripheral intravenous cannulas. Ampicillin, Gentamicin, Ceftriaxone, Metronidazole, Cefotaxime, Ceftazidime and Vancomycin were among the most commonly administered antibiotics. About 100 (56.2%) nurses who insert the cannula had less than 2 years of clinical experience, with median and inter quartile range (3 ± 1), ranges from 1 to 28 years, and 143 (80.3%) held degrees. Fifty-four (30.3%) infants were resuscitated with maintenance fluid and 91 (51.1%) were infused continuously (Table 3).

**Table 3:** Health care factors of lifespan of peripheral intravenous cannula among infants admitted in public hospitals of Mekelle city, Tigray, Ethiopia, 2016

Variables	Category	Frequency (%)	Lifespan of PIVC	
			Short lifespan	Long lifespan
Fluid used	Maintenance Fluid	54 (30.3)	30	24
	10% dextrose	53 (29.8)	30	23
	N/S^a^	21 (11.8)	13	08
	Other^b^	10 (5.6)	02	08
Infusion type	Continuous	91 (51.1)	51	40
	Intermittent	47 (26.4)	24	23
Clinical experience (in yrs.)	<2	100 (56.2)	61	39
	3-5	46 (25.8)	14	32
	6-10	10 (5.6)	08	02
	11-15	11 (6.2)	05	06
	>15	11(6.2)	06	05
Educational level	Diploma	32 (18)	21	11
	Degree	146 (82)	73	73
Attempt to insert	1	73 (41)	36	37
	2-3	78 (43.8)	40	38
	4-6	27 (15.2)	18	09
Medication type	Antibiotic	141 (79.2)	73	68
	Diuretics	17 (9.6)	06	11
	Corticosteroid	17 (9.6)	05	12

^a^ *N/S* Normal saline, ^b^ *Other* Ringer lactate and Dextrose with water

### IV. Lifespan of peripheral intravenous cannula

The duration of patency of peripheral intravenous cannulas (*n* = 178) ranges from 1 to 96 h for a median and IQR of (30 ± 28). This study revealed that the proportion of short lifespan cannula was 52.8% (94) and of this proportion, 37.6% (67) cannulas were removed within 24 h (Table 4).

**Table 4:** Lifespan of peripheral intravenous cannula among infants admitted in public hospitals of Mekelle City, Tigray, Ethiopia, 2016

Variables	Category	Frequency (%)
Lifespan of PIVC^a^		
	Up to 24 h	67 (37.6)
	Between 24 and 48 h	80 (44.9)
	Between 48 and 72 h	21(11.8)
	Between 72 and 96 h	10 (5.6)

^a^ *PIVC* Peripheral intravenous cannula

### IV. Factors affecting the lifespan of peripheral intravenous cannula

As noted from the result of bivariate and multivariate analysis (Table 5), blood transfusion, pediatric unit, insertion site, reason for removal, clinical experience and corticosteroid were variables which showed significant association with a lifespan of a peripheral intravenous cannula (Table 5).

**Table 5:** Bivariate and multivariable logistic regression analysis result of lifespan and associated factors of peripheral intravenous cannula in Mekelle City Public Hospitals, Tigray, Ethiopia, 2016

Variable	Category	Frequency (%)	Lifespan of PIVC		COR [95% CI]	AOR [95% CI]
			Short lifespan	Long lifespan		
Age	Neonate	57 (32)	35	22	1.67 [0.88,3.175]	
	Infant	121 (68)	59	62	1	
Sex	Female	72 (40.4)	41	31	1.323 [0.72,2.415]	
	Male	106 (59.6)	53	53	1	
Pediatric unit	NICU	57 (32)	35	22	2.06 [1.06,3.998] *	1.89 [0.689,5.23]
	PICU	20 (11.2)	15	05	3.89 [1.31,11.51] *	6.93 [1.56,30.71] **
	PW	101 (56.7)	44	57	1	1
Weight	<1.499	9 (5.1)	05	04	1.495 [0.38,5.862]	0.825 [0.089,7.624]
	1.5-2.499	28 (15.7)	20	08	2.99 [1.215,7.36] *	3.011 [0.597,15.192]
	2.5-3.499	29 (16.3)	18	11	1.96 [0.847,4.521]	
	>3.5	112 (62.9)	51	61	1	1.785 [0.478,6.664]
Sickness	Chronic	20 (11.2)	10	10	0.88 [0.347,2.234]	
	Acute	158 (88.8)	84	74	1	
Removal reason	DCWC	42 (23.6)	09	33	1	1
	Dislodge	63 (35.4)	37	26	5.22 [2.14,12.73] *	8.15 [2.49,26.63] **
	Complication	51 (28.7)	36	15	8.8 [3.39,22.797] *	10.48 [3.08,35.65]**
	Blockage	22 (12.4)	12	10	4.4 [1.44,13.44] *	3.91 [0.889,17.158]
Site of insertion	Scalp	77 (43.3)	48	29	1	1
	Arm	54 (30.3)	13	41	0.192 [0.09,0.42] *	0.126 [0.046,0.349]**
	Hand	29 (16.3)	21	08	1.59 [0.622,4.043]	2.16 [0.662,7.075]
	Leg	18 (10.1)	12	06	1.208 [0.41,3.569]	1.263 [0.312,5.113]
Fluid	No	40 (22.5)	19	21	1	
	Yes	138 (77.5)	75	63	1.316 [0.65,2.663]	
Infusion type	Continuous	91 (51.1)	51	40	1.222 [0.60,2.475]	
	Intermittent	47 (26.4)	24	23	1	
Type of fluid	M/F	54 (30.3)	30	24	1	
	10%	53 (29.8)	30	23	1.043 [0.49,2.239]	
	N/S	21 (11.8)	13	08	1.30 [0.463,3.646]	
	Other*	10 (5.6)	02	08	0.20 [0.039,1.031]	
Medication	No	21 (11.8)	16	05	1	1
	Yes	157 (88.2)	78	79	0.309 [0.108,0.883] *	0.17 [0.37,0.784] **
Blood transfusion	No	161 (90.4)	89	72	1	1
	Yes	17 (9.6)	05	12	0.337 [1.113,1.00]	0.120 [0.028,0.509] **
Venous condition	Visible but not palpable	118 (66.3)	65	53	1	
	Not visible and palpable	60 (33.7)	29	31	0.763 [0.409,1.422]	
Educational level	Diploma	32 (18)	21	11	1.91 [0.859,4.242]	1.322 [0.392,4.463]
	Degree	146 (82)	73	73	1	1
Clinical experience	≤2	100 (56.2)	61	39	1	1
	3-5	46 (25.8)	14	32	0.28 [0.13,0.590]*	0.168 [0.060,0.469] **
	6-10	10 (5.6)	08	02	2.56 [0.52,12.68]	1.41 [0.214,9.312]
	11-15	11 (6.2)	05	06	0.533 [0.152,1.87]	1.27 [0.263,6.131]
	>15	11 (6.2)	06	05	0.767 [0.219,2.69]	0.313 [0.049,1.99]
Antibiotics	No	37	21	16	1	
	Yes	141 (79.2)	73	68	0.818 [0.394,1.69]	
Diuretics	No	161 (90.4)	88	73	1	1
	Yes	17 (9.6)	06	11	0.452 [0.16,1.283]	2.36 [0.554,10.046]
Corticosteroid	No	161 (90.4)	89	72	1	1
	Yes	17 (9.6)	05	12	0.337 [0.113,1.00]	0.164 [0.034,0.793] **
Attempt to insert	1	73 (41)	36	37	0.924 [0.488,1.75]	
	2-3	78 (43.8)	40	38	1	
	4-6	27 (15.2)	18	09	1.90 [0.761,4.744]	

*P value <0.05 in the bivariate analysis, **P value <0.05 in the multivariable analysis

## Discussion

In this study, ninety-four (52.8%) of the infants were with a short cannula lifespan (<30 h), and 37.6% of cannulas failed within 24 h. This is lower than the study finding of Rio de Janeiro and California which was 50 and 45.8% respectively [13, 14]. This difference may be related to smaller sample size, different study setup and background of the patients. A majority (168) of cannulas failed within 72 h. This is inline with the study conducted in Gold Coast Hospital, Australia [15].

In this study, the median lifespan of cannulas was 30 h, comparable with the studies conducted previously in the United States in which lifespan was 33 h [4], but less comparable with the study conducted in India which was 40 and 51 h respectively [3, 16]. Smaller sample size might influence the result along with health service related factors and study design.

Pediatric units showed significant association with the lifespan of peripheral intravenous cannula. Infants admitted to a pediatric intensive care unit had a shorter cannula lifespan compared to those who were admitted in the pediatric ward. Similar findings were also observed in an earlier study conducted in the southwestern United States [17]. This might be due to the reason that infants admitted to pediatric intensive care units were critically ill and were more likely to concurrently require more medications.

The lifespan of the peripheral intravenous cannula was significantly reduced by dislodgement and complication. This finding may be supported due to the reason that as the cannula is foreign to the human body, the presence of a foreign body in the vein stimulates an inflammatory response which disposes the development of thrombus and complication.

The survival time of peripheral intravenous cannula was prolonged with the administration of medication as compared to the survival time of peripheral intravenous cannula in those not receiving medication. Specifically, the administration of corticosteroids was found to prolong the cannula lifespan, possibly due to the corticosteroid associated broad anti-inflammatory effect.

This study showed that the site of peripheral intravenous cannula placement in the arm, in comparison to the scalp, significantly increased the longevity of peripheral intravenous cannula. This is consistent with the study conducted previously in West Coast City, United States (best to match the previous reference by naming a region such as Northeast or Southwest as earlier in the document) and Australia [7, 18]. This may be partly a result of the size of the vein into which it is placed, as also demonstrated in this study.

The lifespan of the peripheral intravenous cannula was also prolonged with the transfusion of blood. Similar findings were observed in earlier studies conducted in India and the Southwestern United States [16, 17]. It was hypothesized that the acidic solutions being infused in the peripheral intravenous cannula may be buffered by the pH of the blood.

In this study, the clinical experience of a nurse who insert the cannula was shown to have a significant effect on the lifespan of peripheral intravenous cannula. Cannulas secured by more experienced nurses stayed secure longer as compared to less experienced nurses.

## Conclusion

In conclusion, untimely removal of peripheral intravenous cannulas was higher in infants. This study also found that the pediatric intensive care unit, clinical experience (35 years), insertion site (arm), reason for removal (dislodge and complication), medications, corticosteroids and blood transfusion were identified as variables that showed a statistical significant association with the lifespan of a peripheral intravenous cannula.

## Abbreviation

AOR: Adjusted odd ratio
CI: Confidence interval
IV: Intravenous
PICU: Pediatric intensive care unit
PW: Pediatric ward
SPSS: Statistical Package for Social Sciences
